# First Report of Ethylenediaminetetraacetic Acid-Dependent Pseudo-Thrombocytopenia in Chile: Prevalence and Laboratory Insights

**DOI:** 10.3390/diagnostics15081050

**Published:** 2025-04-21

**Authors:** Mario Balcázar-Villarroel, Florencia Carmine, Francisco Torrens, Katherine Birditt, Cristian Sandoval

**Affiliations:** 1 TecnoMedic Clinical Laboratory, Puerto Montt 5502901, Chile; 2 Escuela de Medicina, Facultad de Medicina, Universidad de La Frontera, Temuco 4811230, Chile; f.carmine02@ufromail.cl; 3 Institut Universitari de Ciència Molecular, Universitat de València, 46071 València, Spain; torrens@uv.es; 4 Physiology Development and Neuroscience Department, University of Cambridge, Cambridge CB2 1TN, UK; krb56@cam.ac.uk; 5 Escuela de Tecnología Médica, Facultad de Salud, Universidad Santo Tomás, Los Carreras 753, Osorno 5310431, Chile; 6 Departamento de Medicina Interna, Facultad de Medicina, Universidad de La Frontera, Temuco 4811230, Chile

**Keywords:** pseudo thrombocytopenia, EDTA, platelet count, sodium citrate

## Abstract

**Background:** Ethylenediaminetetraacetic acid-dependent pseudo thrombocytopenia (EDTA-PCTP) is defined as a false in vitro decrease in the platelet count performed in the EDTA tube due to the spontaneous formation of platelet aggregates that prevent a correct count in hematological auto analyzers. The frequency of EDTA-PCTP varies depending on the population studied, ranging from 0.01% to 30.0%. In Chile, although the diagnosis of this condition is performed in clinical laboratories, only a few isolated reports have been described. **Objectives**: To determine the prevalence of EDTA-PCTP in a cohort of patients who attended an outpatient clinical laboratory in southern Chile over a period of almost 4 years. **Methods**: A retrospective analysis was conducted using the Laboratory Information System from January 2021 to November 2024 to identify patients with suspected and confirmed cases of EDTA-PCTP. **Results**: The prevalence rate observed was 0.044% (12 out of 27,480). Additionally, we established that platelet count measurement from the citrate tube at 2–5 h post-sampling was comparable to the platelet count from the EDTA/K2 tube at time 0 (*p* > 0.05) in these patients. **Conclusions**: We conclude that a relatively low prevalence of EDTA-PTCP was identified in a population of patients attending an outpatient laboratory in Chile, marking the first report of its kind in our country. Future studies may validate our findings to enhance understanding of EDTA-PTCP, thereby preventing incorrect diagnoses and treatments.

## 1. Introduction

Thrombocytopenia is characterized by a reduction in peripheral blood platelet count, with the clinical threshold often set at <150 × 10^9^/L; however, patients with counts beyond 50 × 10^9^/L are usually asymptomatic. Spontaneous bleeding is uncommon in thrombocytopenia; it occurs more commonly when the platelet count is below 20 × 10^9^/L and is prevalent at levels below 10 × 10^9^/L [1]. A genuine reduction may manifest in several situations, including primary immune thrombocytopenia (PIT), viral and bacterial infections, connective tissue disorders, cardiovascular illnesses, substance abuse, hematologic malignancies, and other etiologies [2]. On the other hand, among the causes of a false decrease in platelet count are ethylenediaminetetraacetic acid-dependent pseudo thrombocytopenia (EDTA-PCTP) [3], presence of giant platelets [4], platelet satellitism [5], and/or platelet phagocytosis [6]. The most frequent cause is EDTA-PCTP, defined as a false decrease in vitro platelet counts performed in the EDTA tube due to the spontaneous formation of platelet aggregates that prevent a correct count in hematological auto analyzers [3].

Since the first description of this phenomenon [7], isolated cases have been described, and the prevalence in different types of patients has been estimated. In this regard, the frequency of EDTA-PTCP varies depending on the population studied, being found between 0.01% and 0.27% in the general population [8,9], between 0.1% and 1.9% in hospitalized patients [10], and between 6.6% and 30.0% among patients investigated for thrombocytopenia [11,12]. The appearance of this phenomenon is explained by the presence of antiplatelet autoantibodies in the IgG, IgM, and/or IgA subclass. Due to the chelating effect of calcium (exerted by the EDTA found on the walls of the sample collection tube), a dissociation of the GPIIb/IIIa heterodimer found in the platelet membrane occurs. In this way, antiplatelet autoantibodies are able to bind to crypto antigens that are only exposed in the dissociated form of glycoprotein IIb, which is now exposed and generates the in vitro platelet aggregation observed in the peripheral blood smear [13]. It has been described that this aggregation, in most cases, is temperature-dependent, being more potent at room temperature than at 37 °C, therefore keeping the samples at this latter temperature can guarantee a correct platelet count in most cases, although it should be taken into account that in approximately 20–35% of them this phenomenon will continue, since it depends on the subclass and activity of the antibody involved [10]. The other important factor that explains the degree of platelet aggregation is the time elapsed between taking the venous sample and processing it in the autoanalyzer, since it has been shown that a decrease in the platelet count can occur from 10 min to 4 h after the sample collection [10].

Although there is a study that showed that EDTA-PTCP was a factor of worse prognosis in patients hospitalized in Japan [14] and another study carried out in Spain related it to autoimmune diseases [15], it has traditionally been described that this phenomenon is not clinically significant nor is it associated with any particular pathology [10]. However, its effective recognition is very important because false thrombocytopenia can determine the establishment of erroneous treatments (such as the beginning of therapy with corticosteroids), unnecessary platelet transfusion, postponement of programmed operations, postponement of discharge in hospitalized patients, delay in the start of anticoagulant and/or fibrinolytic therapy in patients with suspected acute myocardial infarction, discontinuation of heparin therapy, and even splenectomy, added to the degree of stress that can be caused for a patient and his family due to the fact of presenting an analyte outside reference values [3]. In Chile, although this phenomenon is widely diagnosed in most clinical laboratories, few cases have been published in the national literature [16], and, to our knowledge, the estimation of its prevalence in any type of population has not yet been carried out. For this reason, the objective of the present work was to determine the prevalence of EDTA-PTCP in a cohort of patients who attended an outpatient clinical laboratory in southern Chile for a period of almost four years.

## 2. Materials and Methods

A retrospective search was performed in the Laboratory Information System (LIS, ProActive©, Christchurch, New Zealand) of the Tecno-Medic outpatient clinical laboratory located in Puerto Montt, between January 2021 and November 2024, to identify those patients with suspected EDTA-PCTP, according to the accomplishment or one or more of the following criteria recommended by the Croatian Society of Medical Biochemistry and Laboratory Medicine [17]:

1. Decreased platelet count (<150 × 10^9^/L) in the EDTA-dipotassium (EDTA/K2) tube (CML Biotech Ltd., Angamaly, India) processed on the Mindray BC-5380 hematology analyzer (Mindray Medical International Ltd., Shenzhen, China) in patients without clinical signs of thrombocytopenia/thrombocytopathies and without previous platelet counts;
2. A significant reduction in platelet count (delta check ≥ 40%) compared to the patient’s previous result. It should be noted that this rule applies when comparing the results of the platelet count of the same patient obtained at different times (intra-individual biological variability);
3. The presence of alarms associated with platelet and/or leukocyte counts added to an alteration in the platelet histogram.

It is important to mention that the methodology used by the hematological autoanalyzer for the platelet count was electrical impedance, which consists of encompassing variation in electric current intensity when a blood particle passes through two electrodes. This technology is used by the majority of clinical laboratories as a consequence of the larger availability of instruments based on this standard technology in the market [18]. Through this measurement, the analyzer generates a Gaussian bell-shaped graph, which combines the platelet count and volume. Any deformation in the typical shape of this graph was considered an alteration of the histogram.

Those patients who met one or more of the criteria previously specified and in whom platelet aggregates were detected, defined as the grouping of at least 5 platelets together [19] and observed according to the ICSH guidelines [20], were called for a second sample collection, this time obtaining two tubes: one tube with EDTA/K2 and one tube with 3.2% sodium citrate (CML Biotech Ltd., Angamaly, India). On the other hand, patients with no observation of platelet aggregates were confirmed as true thrombocytopenia.

**Figure 1:**
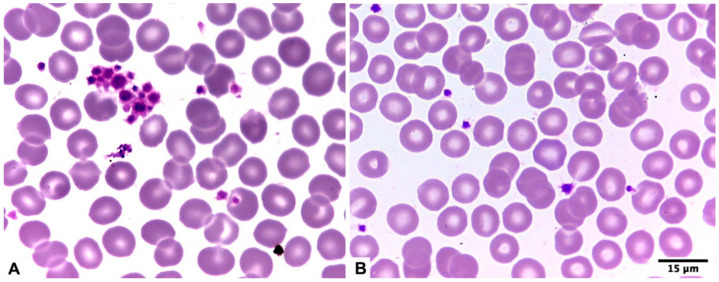
Platelet aggregation in a blood smear from a patient with EDTA-induced pseudo thrombocytopenia (**A**) as compared to a smear prepared from sodium citrate-anticoagulated blood from the same patient (**B**).

Once preanalytical factors related to sample collection were excluded (such as in vitro activation of coagulation due to poor homogenization of the tube and/or clot formation due to difficult puncture), the following procedure was followed: 1. Processing of both tubes in the hematology analyzer immediately after the sample was taken (less than 5 min). It should be noted that the platelet count obtained from the tube with sodium citrate was multiplied by a factor of 1.1 due to the diluting effect of this liquid anticoagulant [19]; 2. Reprocessing of both tubes in the hematology analyzer 2–5 h after the sample was taken (kept at room temperature using a digital thermometer and air conditioner to avoid temperature fluxions due to weather conditions); and 3. Performing a peripheral blood smear, according to the above, 2–5 h after the sample was taken in both tubes for the targeted search for platelet aggregates (Figure 1).

Confirmation of EDTA-PCTP: To define a case as EDTA-PTCP, all of the following criteria had to be met [17,21]: 1. A significantly higher platelet count result was obtained in the sodium citrate tube compared to the EDTA/K2 tube in tubes processed 2–5 h after sampling; 2. Observation of platelet aggregates in the smear made from the EDTA/K2 tube at 2–5 h after sampling and not in the sodium citrate tube; 3. Absence of hemorrhagic signs or symptoms; and 4. Significant reduction in platelet count in the EDTA/K2 tube when comparing the tube processed immediately after sampling with the EDTA/K2 tube processed 2–5 h later.

Despite the criteria mentioned above, for those patients who came from external sampling rooms, more than 46 km away, only criteria 1, 2, and 3 were considered, which are sufficient to make a confirmatory diagnosis of EDTA-PCTP according to the Croatian Society of Medical Biochemistry and Laboratory Medicine [17], since the platelet counts in tubes with EDTA/K2 and sodium citrate could not be processed immediately after the sample collection due to the aforementioned distance.

To determine statistically significant differences between platelet counts performed at different times and in different tubes, the 2-tailed, paired student *t* test was used, with a *p*-value < 0.05 to be considered significant, using using the latest version of the online software GraphPad (https://www.graphpad.com/quickcalcs/ttest2/; last accessed 14 April 2025).

## 3. Results

Of a total of 27,480 complete blood cell counts processed in the clinical laboratory between January 2021 and November 2024, there were 12 patients who met the criteria previously indicated, so the prevalence determined was 0.044% (12/27,480). Table 1 shows that the average age of patients confirmed with EDTA-PCTP was 41.8 years, with a standard deviation (SD) of 22.1 years and a range of 12–81 years. In addition, there was a notable predominance of women over men (10 female and 2 male patients).

Table 2 shows the average platelet counts in both tubes and at different processing times. In this sense, as expected, there was a notable difference between the platelet count performed in the tube with EDTA/K2 at time 0 and the same tube at 2–5 h (*p* < 0.001; statistically significant differences). On the other hand, there were no differences between the platelet count in the tube with sodium citrate at both times (*p* = 0.1401; statistically non-significant differences); nor between the tube with EDTA/K2 and sodium citrate both at time 0 (*p* = 0.6997; statistically non-significant differences); nor between the tube with EDTA/K2 at time 0 and the tube with citrate at 2–5 h (*p* = 0.2666; statistically non-significant differences).

**Figure 2:**
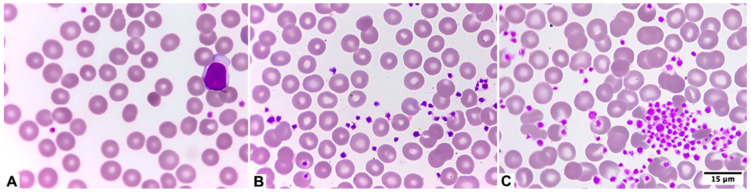
Visual assessment of pseudo-thrombocytopenia in blood smear using EDTA. (**A**). No aggregates. (**B**). Moderate aggregates (++). (**C**). Severe aggregates (+++).

**Figure 3:**
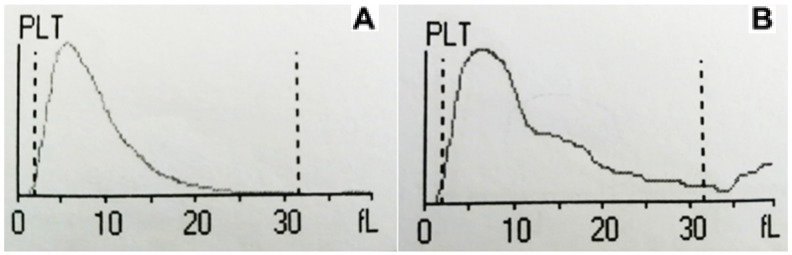
Example of platelet histogram changes over time in the EDTA/K2 tube in patient #3. Note the change in the shape of the graph between tube A and tube B, shifting from the typical bell-shaped curve to a clearly distorted curve because of the presence of an abundance of “large platelets” with sizes over 10 fL. This abnormal platelet histogram alone can be used as a quick clue to the screening and/or diagnosis of EDTA-pseudothrombocytopenia. (**A**). EDTA/K2 0 h. (**B**). EDTA/K2 3 h.

Visual assessment of pseudo-thrombocytopenia in blood smear using EDTA can be seen in Figure 2 and its correlation with platelet histogram changes over time in an EDTA tube can be observed in Figure 3.

## 4. Discussion

EDTA-PTCP is considered an infrequent phenomenon during the daily routine of a clinical laboratory. However, correct identification is essential to avoid unnecessary procedures and/or the initiation of erroneous treatments [3]. Considering its greater frequency among patients with thrombocytopenia, several guidelines have included the investigation of EDTA-PTCP in the case of a new diagnosis of PIT, recommending the observation of the peripheral blood smear by an experienced microscopist, especially when there is no correlation between the platelet count and patient’s symptoms [22,23].

Regarding the prevalence determined in our population (0.044%), this rate is similar to that determined in a hospital in Venezuela (0.035%) [24] and is exactly the same as that reported in a hospital in China (0.044%) [25]. On the other hand, our prevalence was higher than that reported in the Polish population (0.013%) [8], but lower than that reported in Italy (0.068%) [26], Canada (0.231%) [27], Israel (0.272%) [9], and Pakistan (0.278%) [28]. These differences can be explained by the different types of populations studied since it has been reported that the frequency of EDTA-PTCP is higher in hospitalized patients compared to outpatients [10]. On the other hand, the platelet count cut-off point under which EDTA-PTCP research is performed is highly variable among the different published studies (Table 3). This situation causes a wide variability in the degree of screening of patients with EDTA-PTCP, which affects the determined prevalence since a priori it is impossible to know if a patient has this condition, and it is known that the time that elapses between blood sample collection and the processing of the samples in the hematological analyzer can be very variable depending on the daily routine of each clinical laboratory, and, in addition, the time from when platelet aggregation begins to appear in the tube with EDTA can be as variable as 1 min to 4 h [10]. For this reason, we consider it reasonable to use a higher cut-off point for the investigation of EDTA-PTCP (<150 × 10^9^/L) in order to increase the sensitivity in the screening, knowing that it has been shown that up to 20% of patients with EDTA-PTCP have platelet counts between 100 and 149 × 10^9^/L [9].

Another variable that explains heterogeneity in the frequency of patients with EDTA-PTCP is the lack of international guidelines and/or expert committees that allow standardizing laboratory procedures for the suspicion and subsequent confirmation of patients with this condition. Despite the above, during the last years, several efforts have been made in this regard, through guidelines published by researchers [21] and national scientific organizations, such as the French-Speaking Cellular Hematology Group (GFHC) [19] and the Croatian Society of Medical Biochemistry and Laboratory Medicine (CSMBLM) [17]. In this sense, together with a knowledge of our geographical and work context, we believe it is reasonable to adopt this last guide, considering the simplicity of its suspicion and confirmation protocol, even though we would recommend the use of a higher cut-off point (150 × 10^9^/L instead of 100 × 10^9^/L). Another advantage of this protocol is that it only requires the following criteria for confirmation: 1. A significant increase in the platelet count in the citrate tube compared to the EDTA tube; 2. Presence of platelet aggregates in the smear from the EDTA tube; and 3. The absence of platelet aggregates was observed in the smear from the citrate tube [17]. For this reason, in some cases, it would not be necessary to collect a second sample, since if a tube with sodium citrate is available (for coagulation studies, for example), a comparison can be made between this platelet count and that of the tube with EDTA, and also a smear can be made from the tube with citrate to verify the absence of platelet aggregates, in order to have all the information necessary to confirm the EDTA-PTCP. However, it should be noted that in up to 10% to 20% of patients with EDTA-PTCP platelet aggregates may be present in the smear in both tubes, thus confirming a pseudo thrombocytopenia induced by both EDTA and sodium citrate [17], a fairly rare situation that can be resolved by different ways, such as the use of alternative anticoagulants (such as lithium heparin, disodium oxalate, hirudin, magnesium sulfate) [33], determination of the platelet count by fluorescence [34], and/or tube supplementation with kanamycin [24]. However, most of these options are usually not commercially available and/or they are too expensive, especially for developing countries or places with limited resources, which is probably why the CSMBLM recommends performing platelet counting (by the Fonio method) on peripheral blood smears taken directly from capillary blood by finger prick [17], which is a cheaper, easier, and therefore more widely available method. It should be noted that in our series of patients this phenomenon, called by some authors as multi-coagulant pseudo thrombocytopenia [35,36], did not occur, and, although there was a slight decrease in the platelet count performed in the citrate tube at 2–5 h compared to the EDTA/K2 count at time 0 (Table 2), this decrease was not statistically significant (*p* = 0.2666); consequently, we can conclude that the determination of the platelet count from the citrate tube at 2–5 h is equivalent to the platelet count from the EDTA/K2 tube at time 0, results that agree with those reported by Ghali et al. [37]. However, it is worth saying that it has been recommended to increase the traditional dilution factor of the citrate tube (1.1) to a higher one (1.17), especially in samples processed more than 3 h after sampling [38], although further studies would be needed for the definitive adoption of this new factor in clinical laboratories. Based on the above, we can recommend that a repeat complete blood count, performed within a short time after collection, is sufficient to obtain an accurate platelet count. If a delay is inevitable, collecting the blood in a citrate tube and the test to be performed within 5 h will also solve the problem. A normal platelet count obtained from the second sample and the absence of platelet aggregates in slides made by citrate tube proves the presence of pseudothrombocytopenia in the first sample (which has already been defined by the screening procedures), whereas a persistent thrombocytopenia in both tubes would suggest alternative diagnoses and management.

It is also important to point out the limitations of our study, the most obvious being that our series represented a relatively low number of cases (*n* = 12) compared to other studies with a higher number of processed complete blood cell counts, a higher number of patients confirmed with EDTA-PTCP, and/or a longer follow-up time [9,15,25,26]. However, to our knowledge, this is the first report of EDTA-PTCP prevalence in our country, since only individual cases have been described so far [16]. In this sense, our study adds to other prevalence reports in South America, such as those carried out in Venezuela [24], Argentina [31], and Brazil [32] (Table 3). On the other hand, and although it may not be considered a limitation for the objective of our study, we consider it important to point out the absence of a search for antiplatelet antibodies in the serum of patients confirmed with EDTA-PTCP due to the unavailability of this determination in our environment, which, although it does not confirm this condition, does allow establishing the subclass of immunoglobulin involved (which may be, in order of frequency, IgM, IgG, IgA, or a mixture of two immunoglobulins) [10]. Despite the above, several studies have reported that not in all cases of EDTA-PTCP can the presence of these antibodies be demonstrated, a situation that varies between 17.0% and 69.9% of cases [15,29]. Finally, although neither could it be considered a limitation to the fulfillment of the objective of our study, we consider it important to point out the lack of follow-up of patients to determine the permanence or transience over time of EDTA-PTCP, especially after an episode of acute infection, an autoimmune crisis, and/or due to the natural clearance of antiplatelet autoantibodies. In this regard, several cases of EDTA-PTCP with post-infectious presentation have been described in contexts such as COVID-19 pneumonia [39], infectious mononucleosis [40], and sepsis [41], that have persisted, even for months, remitting after recovery from the condition and returning to stable platelet counts in the EDTA tube.

## 5. Conclusions

We can conclude that we determined a relatively low prevalence of EDTA-PTCP in a population of patients who attended an outpatient laboratory in southern Chile, which, to our knowledge, was the first report in our country. Future research, especially prospective and multicenter studies, may confirm or discard our results in order to give them more statistical consistency and increase knowledge in our environment about EDTA-PCTP and, in this way, avoid erroneous diagnoses and/or treatments.

## Figures and Tables

**Table 1:** Clinical characteristics and platelet count from patients diagnosed with EDTA-PCTP.

N°	Gender	Age (Years)	EDTA/K2 0 h (×10^9^/L)	Citrate 0 h (×10^9^/L) *	EDTA/K2 2–5 h (×10^9^/L)	Citrate 2–5 h (×10^9^/L) *	Reason for Consultation
1	Female	60	223	227	69 (4.0 h)	272	Control post COVID-19
2	Male	18	237	254	96 (2.5 h)	193	Dermatological treatment for acne
3	Male	16	214	223	80 (3.0 h)	150	Patient hospitalized awaiting discharge due to PIMS due to COVID-19
4	Female	12	NA	NA	45 (2.0 h)	198	Developmental delay
5	Female	26	292	276	73 (2.0 h)	202	Thrombocytopenia without symptoms
6	Female	31	NA	NA	91 (2.0 h)	303	Preventive health control
7	Female	66	269	284	69 (2.0 h)	198	Thrombocytopenia without symptoms
8	Female	49	329	339	137 (2.0 h)	276	Thrombocytopenia without symptoms
9	Female	53	226	256	54 (2.0 h)	168	Thrombocytopenia without symptoms
10	Female	55	182	176	38 (2.0 h)	134	Thrombocytopenia without symptoms
11	Female	34	NA	NA	105 (5.0 h)	278	Preventive health control
12	Female	81	180	199	72 (2.5 h)	175	Control for Parkinson’s disease and hypertension

NA: Not available. * Platelet counts in citrate tubes are already multiplied by dilution factor 1.1.

**Table 2:** Evaluation of differences between platelet counts.

	EDTA/K2 0 h	Citrate 0 h	EDTA/K2 2–5 h	Citrate 2–5 h	*p* ^a^	*p* ^b^	*p* ^c^	*p* ^d^	*p* ^e^
Mean ± SD (×10^9^/L)	239.1 ± 49.5	248.2 ± 48.8	77.4 ± 27.3	212.2 ± 55.9	<0.001	0.1401	0.6997	0.2666	<0.001

SD: standard deviation; ^a^: comparison between EDTA/K2 0 h and EDTA/K2 2–5 h; ^b^: comparison between sodium citrate 0 h and sodium citrate 2–5 h; ^c^: comparison between EDTA/K2 0 h and sodium citrate 0 h; ^d^: comparison between EDTA/K2 0 h and sodium citrate 2–5 h; ^e^: comparison between EDTA/K2 2–5 h and sodium citrate 2–5 h.

**Table 3:** Frequency of EDTA-PTCP in different populations together with the cut-off used for screening.

References	Country	Total Sample Size	Number and Frequency of Cases (*n*/%)	Platelet Count Cut-Off for Screening (×10^9^/L)
[8]	Poland	76,498	10/0.013%	<100
[9]	Israel	36,780	100/0.272%	<150
[13]	China	55,000	49/0.089%	<100
[24]	Venezuela	25,390	9/0.035%	<150
[25]	China	190,940	84/0.044%	<80
[26]	Italy	33,623	23/0.068%	<160
[27]	Canada	1300	3/0.231%	<150
[28]	Pakistan	1800	5/0.278%	<100
[29]	Italy	430,000	112/0.026%	<150
[30]	Netherlands	45,000	46/0.102%	<150
[31]	Argentina	7403	4/0.054%	<150
[32]	Brazil	28,435	56/0.197%	<150

## Data Availability

The original contributions presented in this study are included in the article. Further inquiries can be directed to the corresponding author(s).
